# Age Effects on Mediolateral Balance Control

**DOI:** 10.1371/journal.pone.0110757

**Published:** 2014-10-28

**Authors:** L. Eduardo Cofré Lizama, Mirjam Pijnappels, Gert H. Faber, Peter N. Reeves, Sabine M. Verschueren, Jaap H. van Dieën

**Affiliations:** 1 MOVE Research Institute Amsterdam, Faculty of Human Movement Sciences, VU University Amsterdam, Amsterdam, The Netherlands; 2 College of Osteopathic Medicine, Michigan State University, East Lansing, Michigan, United States of America; 3 Department of Rehabilitation Sciences, Faculty of Kinesiology and Rehabilitation Sciences, Katholieke Universiteit Leuven, Leuven, Belgium; University of Leicester, United Kingdom

## Abstract

**Background:**

Age-related balance impairments, particularly in mediolateral direction (ML) may cause falls. Sufficiently sensitive and reliable ML balance tests are, however, lacking. This study is aimed to determine (1) the effect of age on and (2) the reliability of ML balance performance using Center of Mass (CoM) tracking.

**Methods:**

Balance performance of 19 young (26±3 years) and 19 older (72±5 years) adults on ML-CoM tracking tasks was compared. Subjects tracked predictable and unpredictable target displacements at increasing frequencies with their CoM by shifting their weight sideward. Phase-shift (response delay) and gain (amplitude difference) between the CoM and target in the frequency domain were used to quantify performance. Thirteen older and all young adults were reassessed to determine reliability of balance performance measures. In addition, all older adults performed a series of clinical balance tests and conventional posturography was done in a sub-sample.

**Results:**

Phase-shift and gain dropped below pre-determined thresholds (−90 degrees and 0.5) at lower frequencies in the older adults and were even lower below these frequencies than in young adults. Performance measures showed good to excellent reliability in both groups. All clinical scores were close to the maximum and no age effect was found using posturography. ML balance performance measures exhibited small but systematic between-session differences indicative of learning.

**Conclusions:**

The ability to accurately perform ML-CoM tracking deteriorates with age. ML-CoM tracking tasks form a reliable tool to assess ML balance in young and older adults and are more sensitive to age-related impairment than posturography and clinical tests.

## Introduction

It is widely accepted that, in our aging society, falls and fall-related injuries are a major problem with high personal and economic impact [1]. Balance impairments form one of the main risk factors for falls, not only in patient populations but also in community-dwelling older adults [2]. Most of the individuals older than 60 years exhibit some degree of balance impairment, which gradually affects mobility and increases dependency [3]. Therefore, early and adequate assessment of balance impairments is of paramount importance to identify those individuals in need of preventive care [4] and to monitor effects of preventive interventions [5].

Mediolateral (ML) balance impairments have in particular been associated with an increased risk of falling in the older population [6]–[8]. For instance, in prospective and retrospective studies, postural sway parameters in the ML direction have been shown to be higher (i.e. larger area and excursion of the centre of pressure) in fallers than in non-fallers [7]. Nevertheless, as balance control declines gradually with aging, current clinical tools are not sensitive enough to detect early stage impairments in community-dwelling older adults, as these tests exhibit ceiling effects [5]. For instance, Berg and POMA scales have shown ceiling effects even in older adults who exhibit moderate to severe limitations of function (i.e. inability to climb stairs without assistance) [9]. Also conventional posturography, does not consistently discriminate between young and older adults [10]. It appears that ability of balance performance measurements to predict fall risk can be improved over that of conventional posturography by adding a more dynamic component, which involves center of mass (CoM) movements or weight shifting [11]. In line with this, slow lateral stepping responses have been associated with fall risk in older adults [6] and based on videos of real-life falls, inadequate weight shifting accounted for 41% of the falls [12]. Although the latter study focused on older adults living in long-term care facilities, previous studies in community-dwelling older adults also suggest that a considerable proportion of falls can be attributed to incorrect weight-shifting or daily-life tasks that challenge ML balance [13], [14].

Sufficient sensitivity to detect age-related impairments in ML balance control, even in relatively fit and healthy community-dwelling older, can be reached by utilizing tests with incremental difficulty, which can probe the limits of the responsiveness of the balance control system in relation to the demands of the task. The responsiveness can be expressed as control bandwidth, i.e. the range of frequencies over which one can operate within some tolerated error level. For example, a low frequency sinusoidal target signal can be tracked closely, but as the frequency of the signal increases, limits in control bandwidth result in growing tracking errors. Bandwidth of ML balance control can be reduced by slower central and peripheral processing of sensory information [15] and reduced ability to execute motor commands due to muscle weakness (reduced strength and power) [16].

Recent work by our group showed that a mediolateral balance assessment task (coined MELBA), using the center of pressure (CoP) for tracking a visual target allows determining limits in control bandwidth even in healthy young adults [17]. In the current study, we used a modified version of MELBA, in which the subject tracks a target with his or her body CoM, instead of CoP. We believe that using CoM instead of CoP is more meaningful and intuitive, since the CoM is the controlled variable in balancing and weight shifting [18].

The aim of this study was to determine the effect of age on balance responsiveness (control bandwidth) using MELBA. We hypothesized that older adults would have a narrower control bandwidth than young adults. To compare sensitivity of MELBA with conventional methods, we also used posturography. In addition, we investigated test-retest reliability of the modified MELBA. Based on results obtained with CoP tracking [17], we hypothesized that test-retest reliability would be similar or better than CoP-tracking.

## Methods

### Participants

Nineteen healthy older and 19 healthy younger subjects were recruited for this study. To further characterize the older participants, the mini mental state examination MMSE, the Quickscreen (QS) [19], short physical performance battery (SPPB) [20], Berg balance scale (BBS) [21], miniBEST (MB) [22], performance-oriented mobility assessment balance section (POMA-B) [23] and timed up-and-go (TUG) [24] were used. Performance during the timed up-and-go with dual task (DTUG) was extracted from the MB. This research was approved by the Ethical Committee of the Faculty of Human Movement Sciences, VU University, Amsterdam (2011-48M), in accordance with the ethical standards of the declaration of Helsinki. All participants were informed of the experimental procedures and signed informed consent was obtained prior to the experiment.

### Task and Procedure

**Figure 1:**
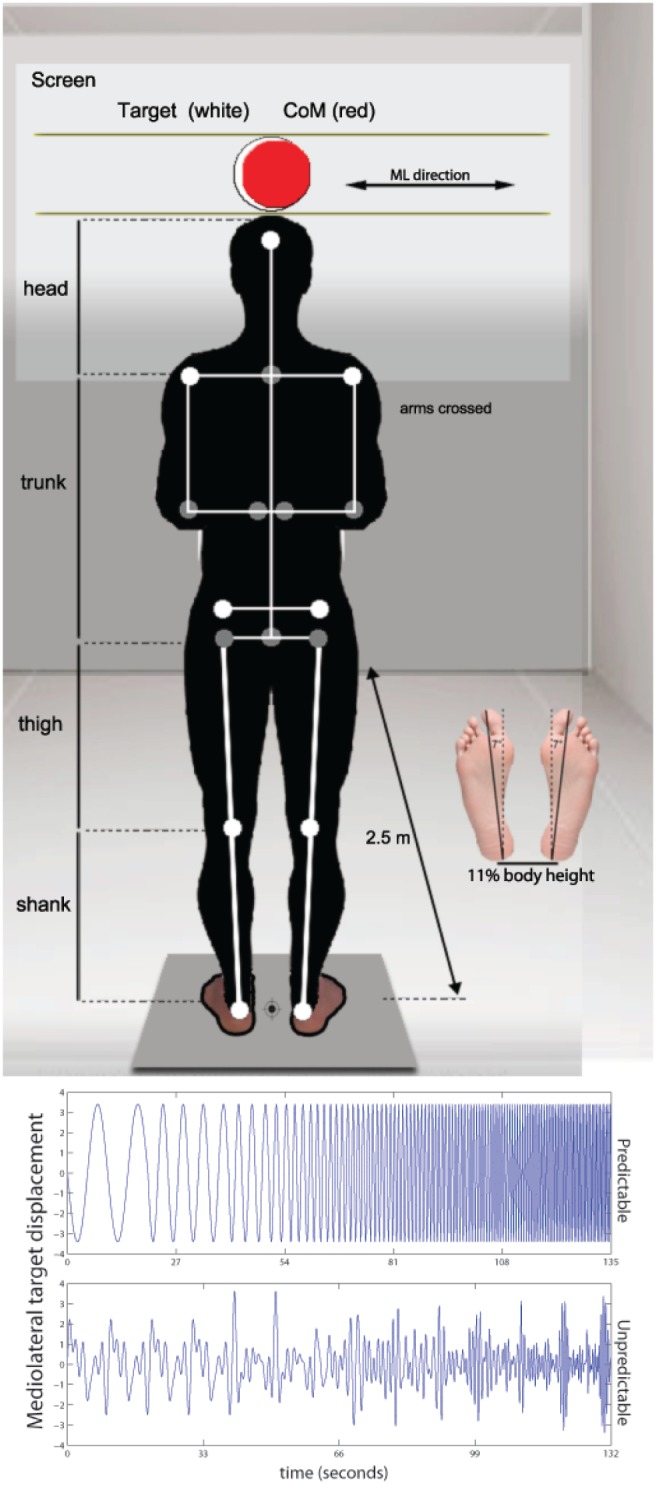
Illustration of the set-up and the model for Center of Mass (CoM) calculation utilized in this experiment, showing a silhouette of a subject standing in the middle of a forceplate with marker placement superimposed (in white actual makers and in grey estimated joint centers) and the display of the CoM feedback (red sphere). The white sphere in the centre represents target which moved in the mediolateral (ML) direction following the patterns depicted in the bottom panel: predictable (top) and unpredictable (bottom). An insertion of foot soles is presented showing foot positioning during the experiments (stance width and angle).

Each participant performed a series of ML-CoM tracking tasks, while standing barefoot and with the arms crossed in a quiet and low-intensity lit room (for set-up details, see Figure 1). Body CoM was calculated with a 9-markers frontal plane model (forehead, shoulder, anterior-superior iliac spines, knees and ankles) using an Optotrak Certus motion capture system (Northern Digital Instruments, Canada). Gender specific CoM calculations were performed using scaling of anthropometric data and inertial parameters described by de Leva [25]. D-flow 3.10.0 software (Motek Medical, The Netherlands) was used to produce target signals as well as to record (60 samples/s) and display target and CoM data on a screen 2.5 m in front of the participant. ML-CoM tracking consisted of tracking a predictable and unpredictable target signal using the ML displacement of the CoM projected on the screen. The target signal and CoM were represented by white and red spheres of 11 and 9 cm diameter, respectively. CoP data were collected using a Kistler-9281B force plate (Kistler Instruments AG, Winterthur, Switzerland) sampled at 60 samples/s.

The *predictable* target signal was constructed using 2 blocks of 20 seconds, 1 block of 10 seconds and 17 blocks of 5 seconds, each composed by one sine wave, which increased in frequency from 0.1 to 2.0 Hz in steps of 0.1 Hz. This information was enhanced using a metronome synchronized with the maximum displacement of the target in order to increase sensory input abundance. The total ML-CoM tracking time for this target signal was 135 seconds.

The *unpredictable* target signal was constructed using 15 blocks composed by the sum of 6 consecutive sine waves separated by 0.1 Hz. A pseudorandom phase-shift between sine waves between –1 to 1 period was introduced in order to avoid predictability. After each block the lowest frequency, which started at 0.1 Hz, was increased by 0.1 Hz until it reached 1.5 Hz. Duration was 40 s for block 1, 20 s for block 2, 10 s for block 3, 8 s for blocks 4 and 5, 6 s for blocks 6 and 7, and 4 seconds for blocks 8 to 15. Duration of the blocks was chosen to obtain a minimum of 2 cycles per frequency contained in the block. The total ML-CoM tracking time for this target signal was 132 seconds. Examples of the two target signals are depicted in Figure 1.

Each participant performed 6 ML-CoM tracking trials: 3 with the predictable and 3 with the unpredictable target. Before performing the test, one practice trial was allowed for each of the conditions. To determine test-retest reliability, all younger and 13 of the older adults repeated the test in a second session 7 days later at the same time of the day. Trials were performed with at least 1 minute of rest in between. Since stance width alters lower limb neuromechanical responses when displacing CoM and CoP in the ML direction [26], stance width was standardized by setting the heel distance to 11% of body height. A fixed 14° stance angle was used across all participants (Figure 1). These stance measures have been shown to be within the values of normal stance [27]. Target maximum side-to-side displacement for both target signals was normalized for each subject at 50% of stance width; allowing ML-CoM displacements to be within the base of support. On average, older participants stood on the force plate with 19.0x±1.0 cm distance between heels, which determined a maximum target displacement of 9.5x±0.5 cm whereas younger participants stood on the force plate with 18.9x±1.1 cm distance between heels, which determined a maximum target displacement of 9.4±0.5 cm. Between groups displacement differences were not significant. Additionally, a subsample of 10 older adults and all younger participants performed 3 standing still trials of 50 seconds with the eyes open and 3 with eyes closed for comparison with ML-CoM non-tracking postural sway measures and conventional posturographic measures (i.e. CoP sway area). No data was discarded and the use of subsamples for the re-test session and posturography measures was imposed by the time constraints of the participants who were unable to attend two sessions.

### Data Analysis

All data analysis was performed using custom-made software in Matlab R2011a (Mathworks, Natick MA, USA). Balance performance over the frequency ranges in the target signal was described by the gain of the linear constant coefficient transfer function between CoM and target signal. This analysis was performed using the Welch algorithm over windows of 0.25 times the length of the target (per block) with 90% overlap between windows [17]. For the unpredictable target, phase-shift, gain and coherence were calculated as the average of the values at each frequency over blocks with overlapping frequency content. The phase-shift (PS) reflects the delay (in degrees) between target and CoM whereas gain (G) reflects the ratio between the target and CoM amplitudes; both in the frequency domain. Perfect performance implies PS = 0 and G = 1 over all frequencies comprising the target signal. In addition, the coherence (Coh) was determined, as a measure of the correspondence between the target and CoM in the frequency domain, which in this study was used to corroborate the assumption of input (target)/output (CoM) linearity and therewith the validity of estimates of PS and G. Perfect linearity produces Coh = 1 over all frequencies comprising the target signal.

To characterize balance performance, 4 descriptors were calculated. First, the values at which PS dropped below 90 degrees and G dropped below 0.5 were determined as the cutoff frequencies (coined f_PS_ and f_G_, respectively). Second, PS_mean_ and G_mean_ were computed as the average of the G and PS values within the bandwidth determined by f_PS_ and f_G_, respectively.

For the posturographic measures (eyes open and eyes closed), CoP sway area and mean velocity, maximal velocity, total excursion and standard deviation of the CoP in the anterioposterior (AP) and ML directions were calculated. Additionally the sum of energies across the .05–2.0 Hz power spectrum of the ML-CoM postural sway was analyzed. This range was chosen since it contains the frequencies present in both targets used in the tracking tasks. Although conventional posturography uses CoP to asses balance, it has been shown that during unperturbed upright standing there is a direct relation between CoP and CoM [18].

### Statistical Analysis

Repeated measures ANOVAs were performed on the dependent variables f_PS_, PS_mean_, f_G_, and G_mean_ with age as a between-subject factor (younger versus older), and target (predictable and unpredictable target) as a within-subject factor. For this analysis the averaged values over three trials performed in session 1 were used. The strength of the age-effect was quantified by calculating the effect size (eta squared).

To analyze test-retest reliability, the data of all subjects participating in both sessions were used. First, to assess systematic differences, a repeated measures ANOVA with age as a between-subject factor (younger versus older), target (predictable and unpredictable target), trial number (1 to 3) and session (1 or 2) as within-subject factors. In view of multiple testing, α was set at .0125 (.05/4). To determine reliability of performance descriptors, intraclass correlations (ICC 2, 1) of the measured variables were calculated for the whole group. To better determine reliability of the measures when applied in a specific age range, ICC was also performed for each age group separately. Measures were considered to exhibit excellent reliability when ICC>.74, good = .60–.74 and fair = .40–.59 [28].

A univariate ANOVA with age as a random factor was performed to determine the effect of age on ML-CoM non-tracking postural sway (conventional posturography). Separate univariate ANOVAs with age as a random factor were used to determine the effect of age on CoP sway measures with eyes open and closed. To better compare age effect on MELBA and conventional posturography, α was also set at 0.0125 and the effect size of age was quantified using eta-squared. Statistical analyses were performed using IBM SPSS (Statistics 21).

## Results

### Subjects

Demographics for all subjects and results of clinical balance tests for the older adults are presented in Table 1. No differences in height and weight were found between groups. Participants did not report any musculoskeletal or neurological condition or use of medication that could affect balance. The older adults scored close to the maximum in all clinical tests and scores were above the cut-off scores for the highest (best balance performance) category defined for each test.

**Table 1:** Top part of the table shows demographics for all participants.

			Older adults		Young	
			mean	sd	mean	sd
Demo-graphics	Age	(years)	72.0	4.6	26.0	3.3
	Height	(m)	1.7	.1	1.7	.1
	Weight	(kg)	76.6	15.2	67.0	12.0
**Clinical measures in Older Adults**						
			**mean**	**sd**	**95% confidence interval**	
time	TUG	(seconds)	6.16	1.05	5.65	6.67
	DTUG	(seconds)	7.29	1.75	6.45	8.13
			**median**			
scores	QS	(min 0)	2		0	4
	BBS	(max 56)	56		53	56
	SPPB	(max 12)	12		10	12
	MiniBEST	(max 28)	26		23	28
	POMA-B	(max 16)	16			

Bottom part of the table shows the descriptive statistics (mean, ± sd, median, lowest and highest scores) for the clinical measures of balance in the older participants: Quickscreen (QS), short physical performance battery (SPPB), Berg balance scale (BBS), miniBEST test (MB) and performance-oriented mobility assessment balance section (POMA-B). For the timed up-and-go (TUG) and dual-task timed up-and-go (DTUG), the mean ± sd and 95% confidence interval are presented.

### Ml-Com Tracking

**Figure 2:**
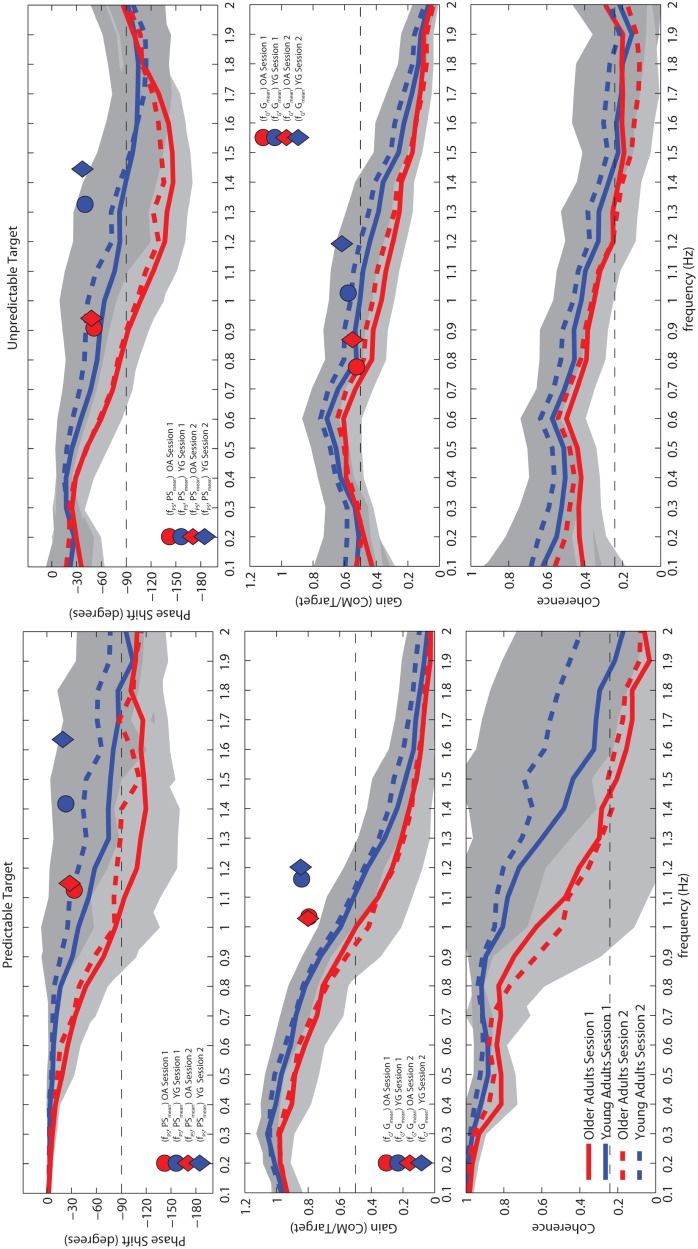
Averaged curves (± sd) for phase shift (top panel), gain (mid panel) and coherence (bottom panel) measures using both, predictable target (left) and unpredictable (right) targets, during first (continuous line) and second (dashed line) sessions and for the younger (in black) and the older adults (in dark grey). Grey shading indicates the ± sd for all subjects and for all trials. Markers inserted in the plots indicate means for performance descriptors for the first session (circular markers) and second session (diamond markers) for the younger (in black) and the older adults (in dark grey).

For all balance performance measures (f_PS_, PS_mean_, f_G_ and G_mean_), significant main effects of age were found (p<.001), indicating a narrower control bandwidth in the older compared to the younger adults (Figure 2; Table 2). In addition, a significant main effect of target was found, with all measures exhibiting lower values when tracking the unpredictable target (Figure 2; Table 2). No interactions between age and target were found. Although lower than for the target main effect, the effect size of age for all measures was medium (η^2^≤0.13) to large (η^2^≤0.38).

**Table 2:** Descriptive statistics for MELBA performance descriptors (f_PS_, PS_mean_, f_G_ and G_mean_) for the predictable and unpredictable targets.

		PREDICTABLE				UNPREDICTABLE				RMANOVA (effects of age and target in 1^st^ session)					
										*p*			η^2^		
		Session 1		Session 2		Session 1		Session 2							
		mean	sd	mean	sd	mean	sd	mean	sd	age	tar	tar*age	age	tar	tar*age
f_PS_ (Hz)	Young	1.42	.34	1.64	.32	1.33	.39	1.45	.34	**<.01**	**<.01**	.11	.26	.35	.08
	Older	1.13	.31	1.15	.33	.91	.17	.94	.20						
PS_mean_ (°)	Young	−23.04	7.08	−19.33	6.77	−40.18	10.07	−37.18	9.21	**<.01**	**<.01**	.72	.36	.87	<.01
	Older	−33.00	5.58	−27.58	5.38	−51.03	6.98	−47.89	9.53						
f_G_ (Hz)	Young	1.16	.10	1.20	.13	1.02	.25	1.19	.27	**<.01**	**<.01**	.14	.34	.45	.07
	Older	1.03	.15	1.03	.16	.77	.15	.87	.14						
G_mean_	Young	.84	.05	.85	.04	.58	.08	.62	.08	**.01**	**<.01**	.83	.20	.92	<.01
	Older	.79	.06	.80	.04	.52	.06	.55	.07						

Right part of the table summarizes the *p*-values and effect sizes (η^2^) of the repeated measures ANOVAs for the between-subjects comparison (‘age’: older vs young adults) the main effect of target (‘tar’: unpredictable and predictable) and age-by-target interaction. Statistically significant *p-*values are presented in bold.

A moderate to high linearity between ML-CoM and the displacement of both targets was found as expressed by mean coherences (0.1 to 2.0 Hz range) >0.4 and >0.6 for unpredictable and predictable ML-CoM tracking, respectively. This supports characterization of balance control using gain and phase-shift. Overall, subjects performed better when tracking the predictable target, reflected by gain values closer to 1 and phase shifts closer to 0, compared to tracking the unpredictable target, especially for input frequencies below 0.8 Hz. For the unpredictable target, near-optimal values for gain and phase were not observed, underlining the challenging nature of this task.

When testing over repeated sessions, significant main effects of session were found for all balance performance measures (all p≤0.01), with a slightly better performance during the second session (Figure 2). Furthermore, we found interactions of session and target for f_G_ and G_mean_ (*p*≤0.01), indicating more improved performance over sessions, when tracking the predictable target. A significant main effect of trial was found only for f_PS_ (*p*<0.01) with a consistent improvement over trials in the younger adults mainly, as indicated by an age-by-trial interaction (*p* = 0.01). A significant interaction of trial and age was also found for PS_mean_ (*p* = 0.01), here with the older adults exhibiting more improved performance over trials. Finally, a significant target-by-trial interaction was found for f_PS_ (*p* = 0.01), with more improved performance over trials when tracking the unpredictable target. In spite of these systematic between-session effects, ICCs showed that for all subjects pooled, reliability of all balance performance descriptors was excellent, with ICC values ranging from 0.77 for G_mean_ when tracking the predictable target to 0.91 for f_PS_ when tracking the unpredictable target (Table 3). As expected, stratified analysis by age group showed lower ICC values, but reliability still ranged from fair to excellent.

**Table 3:** Intraclass correlations (absolute agreement) for the performance descriptors (f_PS_, PS_mean_, f_G_ and G_mean_) for both visual tracking tasks (predictable and unpredictable) for all subjects and stratified by age group.

	Intraclass correlations							
	Predictable				Unpredictable			
	f_PS_	PS_mean_	f_G_	G_mean_	f_PS_	PS_mean_	f_G_	G_mean_
All (32)	**.86**	**.83**	**.86**	**.77**	**.91**	**.88**	**.84**	**.87**
Young (19)	.74	**.83**	.74	.62	**.85**	**.89**	**.78**	**.85**
Elderly (13)	**.95**	.57	**.87**	.64	**.85**	.68	.74	**.85**

Descriptors exhibiting excellent reliability are shown in bold.

### Posturography

No age effect on ML-CoM non-tracking postural sway, as expressed by the energy across the 0.05–2.0 Hz range in quiet standing, was found (younger: 0.27±.09 m^2^/Hz and older: 0.27±.22 m^2^/Hz, *p* = 0.91). In addition, no significant differences were found conventional posturography (CoP sway measures) measures. The largest effect sizes were found for the maximum sway velocity in the ML direction for both, eyes-open and eyes-closed conditions (with p = 0.03, NS after Bonferroni correction), with, however, lower velocities for the older adults.

## Discussion

We studied the effects of age on ML balance control using a ML balance assessment task (MELBA), which consists of tracking predictable and unpredictable visual targets with the body’s CoM. These tasks were used to assess the responsiveness of the balance control system, expressed in terms of control bandwidth. We found a significant effect of age for all descriptors of control bandwidth even though our older participants scored near maximum values on all clinical balance tests. The gradual increase in phase shift and decrease in gain with increasing frequency observed in both groups and for both targets (Figure 2) shows that MELBA tasks are challenging enough to avoid ceiling effects. In contrast, no age effect on ML-CoM postural sway and CoP postural sway during quiet standing were found. The reliability of descriptors of ML balance control bandwidth was also studied. Although small but significant learning effects between sessions, were present, reliability of the descriptors was fair to excellent with ICCs ranging from 0.57 to 0.95.

Although widely used, the evidence for the association of posturographic measures and fall risk in the elderly is inconclusive [29] and age-related changes in postural sway are controversial [10]. In the present study we found overall no age effect and only a trend towards a lower CoP-sway velocity in the ML direction in the older adults. While lower velocity would conventionally be interpreted as reflecting better balance performance, this may be attributed to a reduced exploratory behavior in the older adults, affecting functional variability hence stability [30]. Conversely, it may also reflect the reduced control bandwidth in our older participants revealed by MELBA.

Clinical measures of balance and mobility for older adults were used in the present study, to characterize the subject sample. The near-maximum scores obtained corroborate the ceiling effects reported in community-dwelling older adults [9] and underline that our sample was relatively healthy and fit. For all subjects tested, scores fell within the maximum ranges of the tests. On average, subjects were predicted to have a low risk of falling (QS = 0–1 points [19], BBS = 43–56 points [21]; MB = 19–28 points [31] and TUG and even DTUG<13.5 s [24]), no balance impairments (POMA-B = 14–16 points) [23] and no risk of developing a future disability (SPPB = 10–12 points) [20]. The clinical tests used in this study, are thus not sensitive to subtle impairments of balance that the ML-CoM tracking tasks revealed.

Different factors may account for the lower control bandwidth observed in the older adults. The gluteus medius muscles are strongly involved in ML weight-shifting tasks [32]. When target frequency increases, faster changes in hip torques are required, which could be limited by the rate of force development of the hip abductors [33] possibly due to a selective atrophy of type-II (fast-twitch) fibers [34] and due to a reduced number of fast motor units [35]. Furthermore, tendons become more compliant with age, which can further delay force transmission and thus slow down ML balance responses [36]. It is also plausible that an increased co-activation of antagonist muscles acting in the frontal plane during the tracking tasks may hamper CoM displacement in the ML direction [37], as increased co-activation coinciding with greater stiffness and damping during ML perturbations was found in older adults [38].

In addition to changes at the effector level, impairments of the visual, vestibular, proprioceptive and somatosensory systems may affect balance control. Even though ML-CoM tracking tasks are based on, visual inputs that direct voluntary movements resulting in ML-CoM displacements, accurate online information of CoM position and velocity is needed for execution of accurate motor outputs. Deterioration of the somatosensory system due to aging may provide less accurate proprioceptive information into the balance control system [39]. Proprioceptive impairments due to aging at the hip joint have been reported [40] and may contribute to reducing ML balance control in the older adults. In addition to proprioceptive information, cutaneous plantar receptors and the vestibular organ are involved in providing sensory information into the balance control system even in the presence of explicit visual feedback on CoM movement [41]. Increased perception thresholds of cutaneous plantar receptors with aging have been reported [42] and have been associated with fall risk [19]. Also a reduced function of the vestibular system has been observed with aging [42]. The relevance of this impairment was questioned, because it was not associated with balance impairment as assessed with the POMA [42], but this may be explained by this scale not being sufficiently sensitive, as shown by the results of our study. Effects of decreased vestibular function may be more pronounced when balance is assessed with MELBA, since faster and higher amplitude body movements are made, which would rely more on vestibular information than small-amplitude and slow movements [43].

Multisensory integration is the process by which information arising from different sensory modalities is simultaneously collected [44]. Parallel weighting of sensory inputs occurs in order to control balance according to the demands imposed for a given task. For instance, impairment or absence of a sensory modality causes an up-weighting of other more reliable sources [45]. It has been proposed that the ability to re-weight sensory information as well as to perform parallel cognitive tasks is affected by aging [46],[47]. Inability to properly weight sensory information and altered sensorimotor integration [48] might therefore partially explain the lower balance performance in our older adults. This is in line with previous studies that reported increased processing delays during visuomotor tasks with stepping responses [49]. Similarly, slow reactions during stepping responses have been observed in fallers who exhibited longer gluteus medius onset times [6].

Comparisons between predictable and unpredictable ML-CoM tracking tasks showed a smaller phase shift and higher gain when tracking the predictable target. This may indicate more involvement of cognitive components and more reliance upon feedback mechanisms when performing the unpredictable task [17]. Dual-tasks, used to determine the relationship between cognition and balance and balance-recovery, have shown a decreased balance performance in older adults [47]. This cognition-balance interference could be expected to cause a lower performance in the older adults, especially when tracking the unpredictable target. However, we did not find an interaction of age and task suggesting that other neuro-musculoskeletal factors, as those mentioned above, are more likely to affect ML balance performance than the decline of cognitive resources in the healthy older adults.

Although significant between-sessions differences were found, the ICC values for ML-CoM tracking performance descriptors show these to be reliable measures. All cut-off frequency descriptors (f_PS_ and f_G_) had excellent reliability also in the older adults. This indicates that the bandwidth at which performance is above the thresholds (PS>−90° and G>0.5) highly correlates over sessions. The somewhat lower ICC and higher mean values for PS_mean_ and G_mean_ indicate that, within this bandwidth, performance is more variable, especially for PS and for the predictable target. Compared to the previous version of MELBA, in which CoP instead of CoM feedback was used, reliability was better in the present study, especially for the unpredictable target [17]. This may be due to the fact that ML-CoP tracking is less constrained and could allow different motor strategies, which may vary across trials and between-sessions.

The occurrence of learning effects (except for f_PS_) between, but not within sessions, has previously been interpreted as dissociation between the ongoing learning process and the adaptation after exposure to a novel task [50]. The results partly support the premise that visuomotor processing delay can be improved by training [51]. Although no interaction effects of session-by-age were found, differences in the average ML-CoM tracking performance between the first and second sessions were larger in the younger subjects for all descriptors except PS_mean_, for which improvements were larger in the older adults in both tracking tasks. Overall this indicates that also older adults are able to improve ML balance through training. However, correlations with daily-life ML balance performance using accelerometers should be assessed to explore the relevance of such training effects.

MELBA tasks aim to assess weight-shifting ability, which has been found to be deteriorated and associated to falls in older adults [12]. Performance on the predictable ML-CoM tracking may indicate maximal capacities within the requirements of the task, whereas performance on the unpredictable ML-CoM tracking can give insights into the sensorimotor integration in a more reactive manner [17]. The later may be more associated to stressing situations as those observed when internal or external perturbations are applied. Although the tracking tasks imposed do not simulate daily-life dynamic balance demands, MELBA challenges mediolateral balance control to one’s maximal capacities, thereby yielding highly sensitive outcomes. Further longitudinal research needs, however, to assess the predictive value of ML balance performance on MELBA for fall risk. Finally, the utilization of less expensive and more user-friendly motion capture systems should be explored to simplify MELBA’s setup to make it more clinically available.

## Conclusions

In conclusion, the ability to accurately track predictable and unpredictable targets deteriorates with age. This indicates a deterioration of ML balance in apparently healthy older adults. MELBA appears to be a sensitive and reliable tool to assess ML balance performance in younger and community-dwelling older adults.
