# Correction: Age Effects on Mediolateral Balance Control

**DOI:** 10.1371/journal.pone.0118012

**Published:** 2015-02-03

**Authors:** The PLOS ONE Staff

The third and fourth author's names are spelled incorrectly. The correct names are: Gert S. Faber and N. Peter Reeves. The correct citation is: Cofré Lizama LE, Pijnappels M, Faber GS, Reeves NP, Verschueren SM, et al. (2014) Age Effects on Mediolateral Balance Control. PLoS ONE 9(10): e110757. doi:10.1371/journal.pone.0110757
